# Acute cholangitis due to afferent loop syndrome after a Whipple procedure: a case report

**DOI:** 10.4076/1757-1626-2-6339

**Published:** 2009-08-25

**Authors:** John Spiliotis, Demetrios Karnabatidis, Archodoula Vaxevanidou, Anastasios C Datsis, Athanasios Rogdakis, Georgios Zacharis, Demetrios Siamblis

**Affiliations:** 1 Department of Surgery, Messologi General HospitalMessologi, 30100Greece; 2 Department of Interventional Radiology, University of Patras Medical SchoolRion, Patras 26500Greece; 3 Department of Anesthesiology, Messologi General HospitalMessologi, 30100Greece

## Abstract

**Introduction:**

Patients with resection of stomach and especially with Billroth II reconstruction (gastro jejunal anastomosis), are more likely to develop afferent loop syndrome which is a rare complication. When the afferent part is obstructed, biliary and pancreatic secretions accumulate and cause the distention of this part. In the case of a complete obstruction (rare), there is a high risk developing necrosis and perforation. This complication has been reported once in the literature.

**Case presentation:**

A 54-year-old Greek male had undergone a pancreato-duodenectomy (Whipple procedure) one year earlier due to a pancreatic adenocarcinoma. Approximately 10 months after the initial operation, the patient started having episodes of cholangitis (fever, jaundice) and abdominal pain. This condition progressively worsened and the suspicion of local recurrence or stenosis of the biliary-jejunal anastomosis was discussed. A few days before his admission the patient developed signs of septic cholangitis.

**Conclusion:**

Our case demonstrates a rare complication with serious clinical manifestation of the afferent loop syndrome. This advanced form of afferent loop syndrome led to the development of huge enterobiliary reflux, which had a serious clinical manifestation as cholangitis and systemic sepsis, due to bacterial overgrowth, which usually present in the afferent loop. The diagnosis is difficult and the interventional radiology gives all the details to support the therapeutic decision making. A variety of factors can contribute to its development including adhesions, kinking and angulation of the loop, stenosis of gastro-jejunal anastomosis and internal herniation. In order to decompress the afferent loop dilatation due to adhesions, a lateral-lateral jejunal anastomosis was performed between the afferent loop and a small bowel loop.

## Introduction

The afferent loop syndrome (ALS) is a rare complication of a partial distal gastrectomy with Billroth II reconstruction. We report about a patient who developed ALS, one year after a Whipple procedure that was performed because of a pancreatic carcinoma. The patient suffered two months before the re-admission of recurrent episodes of cholangitis and did develop sepsis secondary to reflux of contents from the afferent loop to the biliary tract via to the biliary jejunal anastomosis. The patient was treated successfully with re-operation.

## Case presentation

A 54-year old Greek man had undergone a pancreato-duodenectomy (Whipple procedure) one year earlier due to a pancreatic adenocarcinoma. The reconstruction was achieved through the sequential placement of pancreatic, biliary and gastric anastomosis into the same jejunum loop. The operation progressed without any complications. Approximately 10 months after the initial operation, the patient started having episodes of cholangitis (fever, jaundice) and abdominal pain. This condition progressively worsened and the suspicion of local recurrence or stenosis of the biliary-jejunal anastomosis was discussed. A few days before his admission the patient developed signs of septic cholangitis. The performed computed tomography (CT) of his abdomen showed neither a biliary- jejunal stenosis nor signs of tumor recurrence. The laboratory evaluation demonstrated malnutrition, elevated white cells count (21000/mm^3^) and elevated bilirubin (14.5 mg/dl).

**Figure 1.**
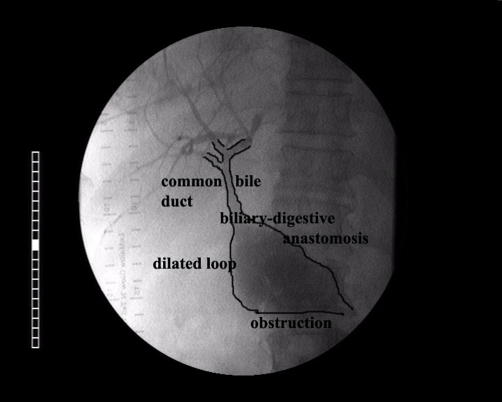
Obstruction of the afferent loop (Percutaneous transhepatic cholangiography).

**Figure 2.**
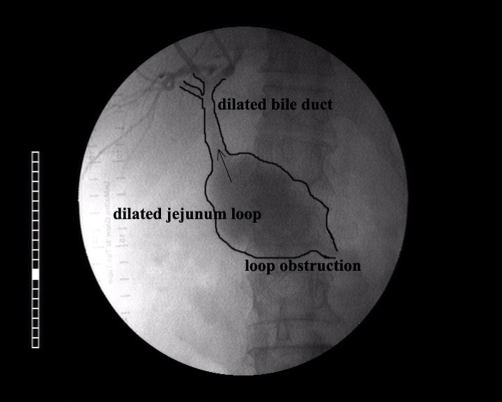
Jejunum-biliary reflux due to elevated loop pressure (Percutaneous transhepatic cholangiography).

A percutaneous transhepatic cholangiography was performed, in order to evaluate the biliary tract anatomy and place a stent in the anastomosis. Based on these findings, a diagnosis of severe ALS with prominent enterobiliary reflux was made (Figure 1 and Figure 2).

During the re-operation we found adhesions in the patient’s upper abdomen and the afferent loop was blocked between the adhesions. A lateral- lateral jejunal anastomosis was performed between the afferent loop and a small bowel loop in order to decompress the afferent loop dilatation.

The patient was discharged on postoperative day 11. In one year follow-up, he was asymptomatic and finally died because of liver metastasis 4 years after the Whipple procedure.

## Discussion

Afferent loop syndrome (ALS) is an unusual complication, with a reporting incidence of 0.2% to 20%, that occurs after Billroth II gastrojejunostomy with partial gastrectomy. ALS is also an extremely rare complication of pancreaticoduodenectomy [1]. Usually it is a chronic complication, and a variety of factors can contribute to its development including adhesions, kinking and angulation of the loop, stenosis of gastro-jejunal anastomosis and internal herniation [2].

When the afferent part is obstructed, biliary and pancreatic secretions accumulate and cause the distention of this part. In the rare case of complete obstruction, there is a high risk of developing necrosis and perforation. This condition is a surgical emergency and requires immediate intervention. In our case the elevated loop pressure developed a reflux of secretions to the intrahepatic biliary tract (Figure 2) and provoked the suppurative cholangitis [3].

Patients who have undergone the Whipple procedure have a fundamental and anatomical configuration of upper abdominal organs than the operation for gastric disorders. The Whipple procedure leaves a combination of an afferent loop of intestine and a biliary-enteric anastomosis, in the case of this patient an end choledocho-jejunostomy was performed as a part of reconstruction after pancreatic resection [4].

This kind of anastomosis permits a reflux without a clinical significance and aerobilia is a normal radiological finding after a biliary-entero anastomosis.

Our case demonstrates a rare complication with serious clinical manifestation of the afferent loop syndrome. This advanced form of ALS led to the development of huge enterobiliary reflux which resulted in cholangitis and systemic sepsis, due to bacterial overgrowth, which usually present in the afferent loop [5]. Only a few papers have been published in the literature regarding this situation. Most of these reported cases of ALS after pancreaticoduodenectomy or gastrectomy which led to repeated episodes of acute pancreatitis with or without jaundice [1,6-9].

The diagnosis is difficult and the interventional radiology gives all the details to support the therapeutic decision making. Some controversy exists regarding the most appropriate treatment. Today non surgical approaches such as external percutaneous or transhepatic drainage and internal drainage by endoscopic stenting are the first choice [10]. However, in some patients a reoperation is needed, and performing a bypass as in our patient we can resolve the obstruction.
